# USP10 is a critical factor for Tau-positive stress granule formation in neuronal cells

**DOI:** 10.1038/s41598-019-47033-7

**Published:** 2019-07-22

**Authors:** Svetlana Piatnitskaia, Masahiko Takahashi, Hiroki Kitaura, Yoshinori Katsuragi, Taichi Kakihana, Lu Zhang, Akiyoshi Kakita, Yuriko Iwakura, Hiroyuki Nawa, Takeshi Miura, Takeshi Ikeuchi, Toshifumi Hara, Masahiro Fujii

**Affiliations:** 1 Division of Virology, Niigata University Graduate School of Medical and Dental Sciences, Niigata, 951-8510 Japan; 2 Department of Pathology, Brain Research Institute, Niigata University, Niigata, 951-8585 Japan; 3 Department of Molecular Neurobiology, Brain Research Institute, Niigata University, Niigata, 951-8585 Japan; 4 Department of Molecular Genetics, Brain Research Institute, Niigata University, Niigata, 951-8585 Japan; 5 Department of Medicinal Biochemistry, School of Pharmacy, Aichi Gakuin University, Nagoya, Japan

**Keywords:** Alzheimer's disease, Mechanisms of disease

## Abstract

Tau aggregates in neurons of brain lesions is a hallmark pathology of tauopathies, including Alzheimer’s disease (AD). Recent studies suggest that the RNA-binding protein TIA1 initiates Tau aggregation by inducing the formation of stress granules (SGs) containing Tau. SGs are stress-inducible cytoplasmic protein aggregates containing many RNA-binding proteins that has been implicated as an initial site of multiple pathogenic protein aggregates in several neurodegenerative diseases. In this study, we found that ubiquitin-specific protease 10 (USP10) is a critical factor for the formation of Tau/TIA1/USP10-positive SGs. Proteasome inhibition or TIA1-overexpression in HT22 neuronal cells induced the formation of TIA1/Tau-positive SGs, and the formations were severely attenuated by depletion of USP10. In addition, the overexpression of USP10 without stress stimuli in HT22 cells induced TIA1/Tau/USP10-positive SGs in a deubiquitinase-independent manner. In AD brain lesions, USP10 was colocalized with Tau aggregates in the cell body of neurons. The present findings suggest that USP10 plays a key role in the initiation of pathogenic Tau aggregation in AD through SG formation.

## Introduction

Tau is a microtubule-associated protein implicated as the causative factor of several neurodegenerative diseases (tauopathies), including Alzheimer’s disease (AD) and a subtype of frontotemporal dementia (FTD)^1,2^. AD is a progressive neurodegenerative disease that impairs memory and other mental functions and it is the most frequent cause of dementia. FTD is also a frequent cause of dementia, affecting the frontal and temporal lobes of the brain. Aberrant Tau protein inclusions in neuronal cells in brain lesions are the pathological hallmark of tauopathies, including AD^1^ and FTD^2^. However, precisely how this pathological Tau aggregation is initiated in AD and FTD processes remains unclear.

The human brain expresses six tau isoforms that are generated by alternative splicings: either zero, one or two amino-terminal inserts (0N, 1N, or 2N) and either three or four repeats of microtubule-binding domains (3R, 4R)^1^. Tau stabilizes microtubules by binding to microtubules, which is its major function. The binding of Tau to microtubules is attenuated by the phosphorylation of Tau at multiple sites^3^. Detergent-insoluble phosphorylated Tau (pTau) has been detected in Tau-positive inclusions of AD brains^4^. Thus, the phosphorylation of Tau is a factor promoting Tau pathologies.

Accumulating evidence suggests that two mechanisms—aggresomes and stress granules (SGs)—initiate Tau aggregation in cultured cells and tauopathies. Aggresomes are inducible large protein aggregates containing many ubiquitinated proteins formed in the cytoplasm of cells treated with a proteasome inhibitor^5^. Such proteasome inhibitor treatment induces ubiquitination of Tau, which is then also localized in aggresomes^6,7^. Aggresome formation is promoted by p62 and HDAC6. P62 is a ubiquitin receptor which binds to ubiquitinated proteins and promotes ubiquitinated protein aggregation^8^. HDAC6 interacts with p62 bound to ubiquitinated proteins and stimulates the transport of many small p62-positive ubiquitinated protein aggregates in the cytoplasm to the perinuclear region to form one big protein aggregate (aggresome)^9^.

SGs are stress-inducible aggregates containing RNA-binding proteins and RNAs that exert protective activities against various stresses, such as the inhibition of apoptosis^10–12^. SGs contain many ribosome-localizing proteins, and SG formation is tightly associated with the suppression of several ribosome-associated functions, including translation^13^. Under certain stress conditions, Tau is localized in SGs in cultured cells^14^. Intriguingly, a recent study suggested that SGs are the initial site for protein aggregation of many neuropathogenic proteins, including Tau^15–21^.

T-cell intracellular antigen 1 (TIA1) is an RNA binding protein with a prion-like aggregation domain that promotes SG formation under various stress conditions^22^. Several pieces of evidence suggest that TIA1 plays a critical role in the aggregation of pathogenic proteins, including Tau, in neurodegenerative disorders, such as AD and FTD, through augmented SG formation^14,15,21^. Vanderweyde *et al*. showed that TIA1 stimulates Tau aggregation and Tau toxicity both in cultured cells and AD model mice by promoting Tau-positive SG formation^14,15^. Ubiquitin-specific protease 10 (USP10) is a member of the ubiquitin-specific protease family, the substrates of which include the tumor suppressors p53 and sirtuin 6^23,24^. Intriguingly, USP10 promotes the formation of both SGs and aggresomes^12,25^. USP10 localizes in SGs in cultured cells treated with several SG stimulators by directly interacting with the SG-initiation protein G3BP, and the depletion of USP10 partially reduces the formation of these SGs^12^. In addition, USP10 depletion also reduces aggresome formation in cells treated with a proteasome inhibitor^25^. Furthermore, by inducing aggresome formation, USP10 augments the aggregation of several pathogenic proteins, such as α-synuclein, a causative factor of Parkinson disease^25^. Based on these previous observations, we decided to investigate whether or not USP10 plays a role in Tau aggregation.

In the present study, we found that USP10 depletion in cultured neuronal cells severely reduced the formation of TIA1/Tau-positive SG formation induced by a proteasome inhibitor or the overexpression of TIA1. USP10 overexpression without proteasome inhibitor treatment also induced TIA1/Tau/USP10-triple-positive SG. Furthermore, USP10 was colocalized with pTau aggregates in brain lesions of AD patients. Thus, the present findings suggest that USP10 plays a role in Tau aggregation during AD development by inducing Tau-positive SG formation.

## Results

### Tau is localized in SGs and aggresomes with USP10

**Figure 1:**
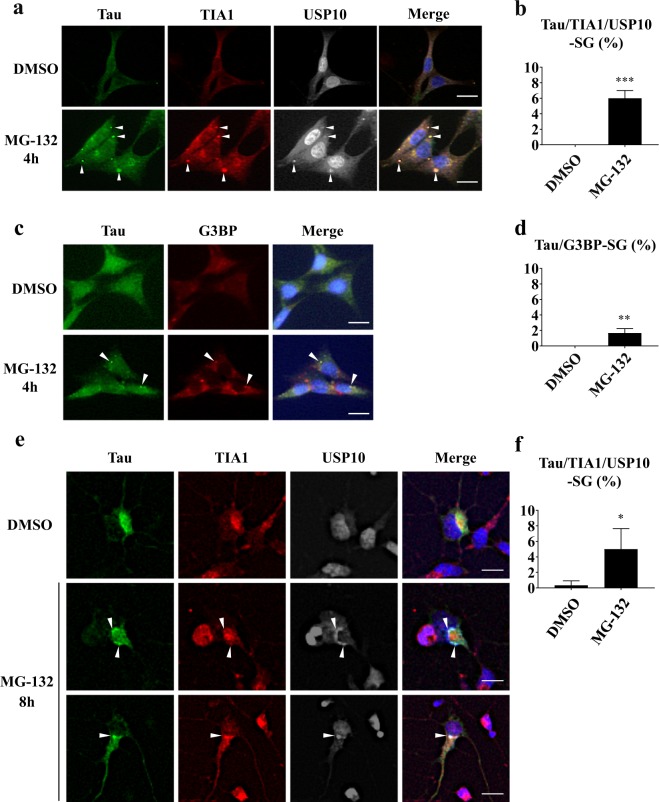
Tau is recruited in SGs in neuronal cells. (**a**–**d**) HT22 cells were treated with 5 µM MG-132 for 4 h and stained with anti-Tau (TAU-5), anti-TIA1, anti-USP10, or anti-G3BP. Nuclei were stained with Hoechst in (**c**). Staining was visualized by a fluorescent microscope. Arrowheads indicate the colocalization of Tau with both TIA1 and USP10 and Tau with G3BP. SG-positive cells were counted as described in the Materials and Methods section, and the population (%) of SG-positive cells is presented as the mean and SD from three independent experiments in (**b**, **d**). **P < 0.01; ***P < 0.001. Scale bar: 10 µm. (**e**,**f**) Rat primary neuron-enriched cells were treated with 2 µM MG-132 for 8 h, and cells were stained with anti-Tau (TAU-5), anti-TIA1 and anti-USP10. SG-positive cells were counted, and the population (%) of SG-positive cells is presented as the mean and SD from three independent experiments in (**f**). *P < 0.05.

Previous studies showed that SGs and aggresomes play key roles in Tau aggregation^14,15,26,27^. To examine the role of USP10 in Tau aggregation, we first examined whether or not Tau is localized in SG with USP10 in neuronal cells. HT22 is a mouse hippocampal neuronal cell line^28^. HT22 cells were treated with MG-132 for 4 h, and SG formation and Tau localization were measured by three SG marker proteins: USP10, TIA1 and G3BP (Fig. 1a–d). Without MG-132 treatment, Tau protein was detected diffusely in the cytoplasm and the nucleus. MG-132-treatment induced multiple Tau aggregates in the cytoplasm, and numerous Tau-aggregates were colocalized both with USP10 and TIA1. These results suggested that Tau is localized in SG with USP10 and TIA1 in HT22 cells treated with MG-132. We called these Tau/TIA1/USP10-positive aggregates Tau/TIA1/USP10-SGs. Tau was also colocalized with G3BP in G3BP-positive SGs (G3BP-SGs), but the colocalization was less marked than that with TIA1 or USP10. PABP, eIF4E and ataxin-2 are RNA-binding proteins. They have been shown to be localized in SGs^29,30^. Tau aggregates were also colocalized with PABP but not with either eIF4E or ataxin-2 (Supplementary Fig. S1). These results suggested that Tau is localized in SGs, with some members of SG-localizing proteins (TIA1, USP10, G3BP and PABP), in HT22 cells. We also examined the colocalization of Tau with USP10 in primary neurons prepared from rat brain. Like HT22 cells, MG-132 treatment of primary neuron-rich cells for 8 h induced Tau/TIA1/USP10-triple-positive SGs (Fig. 1e,f).

Unlike MG-132 treatment for 4 h, MG-132 treatment for 12 h induced Tau/USP10-positive aggresomes in HT22 cells (Supplementary Fig. S2). Tau was also colocalized with TIA1 in aggresomes in HT22 cells treated with MG-132 for 12 h. Taken together, these results suggested that Tau is sequentially localized in SGs and aggresomes in HT22 cells treated with MG-132.

Tau forms insoluble aggregates in the brain lesions of Alzheimer’s disease (AD) patients. We examined the subcellular localization of Tau and SG-associated proteins (USP10, TIA1, G3BP, PABP, eIF4E and ataxin-2) in HT22 cells (Supplementary Fig. S3). Tau is predominantly localized in the NP-40-insoluble fraction in HT22 cells, and the localization was not affected by MG-132 treatment. USP10, TIA1, G3BP and PABP that were colocalized with Tau-SG were detected in the insoluble fraction. These results suggested that insoluble Tau forms SGs in HT22 cells treated with MG-132.

### USP10 knockdown reduces the formation of Tau/TIA1-SGs in HT22 cells

**Figure 2:**
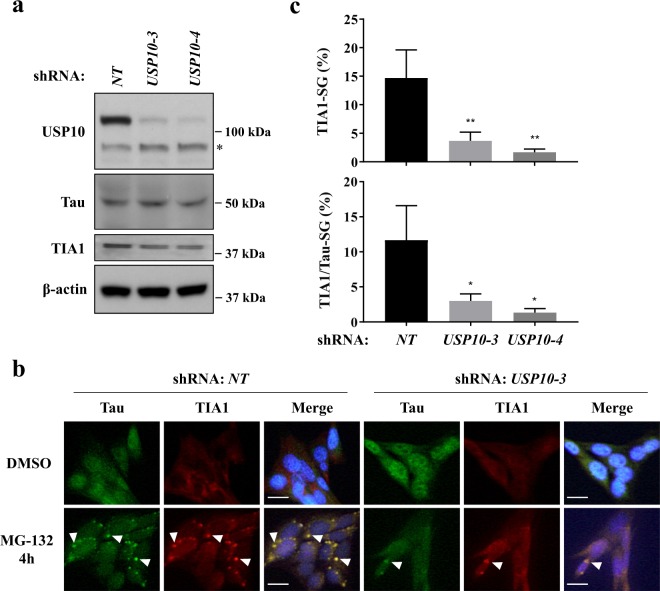
USP10-knockdown reduces Tau/TIA1-SG in HT22 cells. (**a**) Knockdown of USP10 (USP10-KD) in HT22 cells was performed using the lentivirus vector encoding USP10/shRNA (*USP10-3*, *USP10-4*) or the control (*NT*). The expression of USP10, Tau, TIA1 and β-actin in these cells was measured by Western blotting using respective antibodies. The asterisk indicates a nonspecific band. (**b**,**c**) USP10-KD HT22 cells and the control (*NT*) were treated with 5 µM MG-132 for 4 h, and SG formation was evaluated by anti-Tau (TAU-5) and anti-TIA1 antibodies as well as Hoechst for nuclear staining using a fluorescent microscope. Arrowheads indicate the colocalization of Tau with TIA1. The population (%) of cells with TIA1-SGs or TIA1/Tau-SGs is presented as the mean and SD from three independent experiments in (**c**). *P < 0.05; **P < 0.01. Scale bar: 10 µm.

USP10 depletion reduces G3BP1-positive SG formation^12^. To evaluate the role of USP10 in Tau/TIA1-positive SG formation, we established USP10 knockdown (USP10-KD) HT22 cells using USP10 short-hairpin RNA (shRNA). Western blotting showed the reduction of USP10 protein in two USP10-KD cells (*USP10-3*, *USP10-4*) targeting distinct USP10 RNA sequences relative to cells transduced with the control shRNA (*NT*, USP10-WT cells) (Fig. 2a). MG-132 treatment induced TIA1-positive SGs in two USP10-KD cells, but the SG formation was much less marked than that of USP10-WT cells (Fig. 2b,c). These results suggested that USP10 is a factor required for the formation of TIA1-SGs and Tau/TIA1-SGs.

### USP10-KD reduces the TIA1-overexpression-induced SG formation in HT22 cells

**Figure 3:**
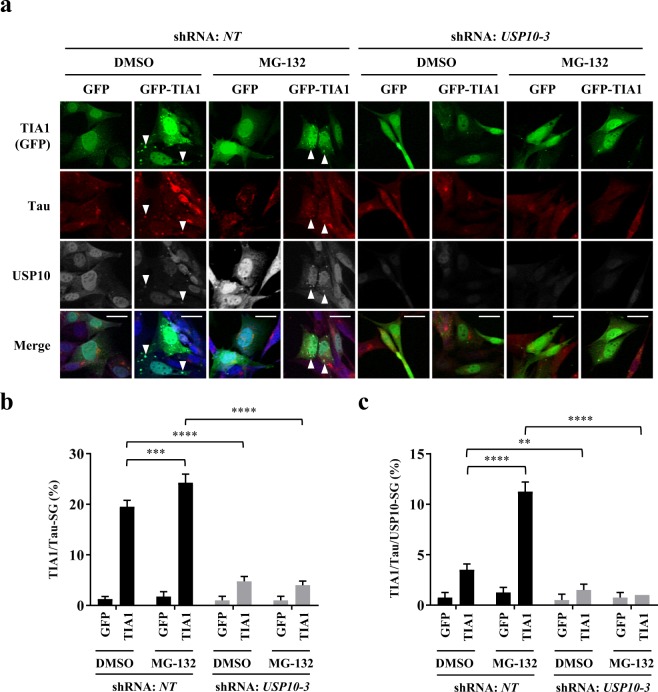
USP10-KD reduced the TIA1-induced Tau/TIA1-SG formation. (**a**–**c**) USP10-KD HT22 cells and the control (*NT*) were transfected with GFP-TIA1 plasmid using lipofection with Lipofectamine 2000. At 48 h after transfection, cells were treated with 5 µM MG-132 for 4 h, and SG formation was evaluated by GFP fluorescence together with anti-Tau (TAU-5) and anti-USP10 staining using a fluorescent microscope. Arrowheads indicate the colocalization of TIA1, Tau and USP10. The population (%) of cells with TIA1/Tau-SG or TIA1/Tau/USP10-SGs is presented as the mean and SD. **P < 0.01; ***P < 0.001; ****P < 0.0001. Scale bar: 10 µm.

Vanderweyde *et al*. showed that the overexpression of TIA1 in HT22 cells induces Tau-positive SG formation^14^. To examine whether or not USP10 is required for TIA1-induced SG formation, we expressed green fluorescent protein (GFP)-tagged TIA1 (GFP-TIA1) in USP10-KD HT22 cells, and cells were treated with MG-132. The overexpression of GFP-TIA1 with or without MG-132 treatment induced GFP-TIA1/Tau-positive SGs in the USP10-WT cells (*NT*), but the induction was severely reduced in UP10-KD cells (Fig. 3a–c). These results suggested that USP10 plays a critical role in TIA1-induced SG formation.

### The overexpression of USP10 induces Tau/TIA1/USP10-SGs in HT22 cells

**Figure 4:**
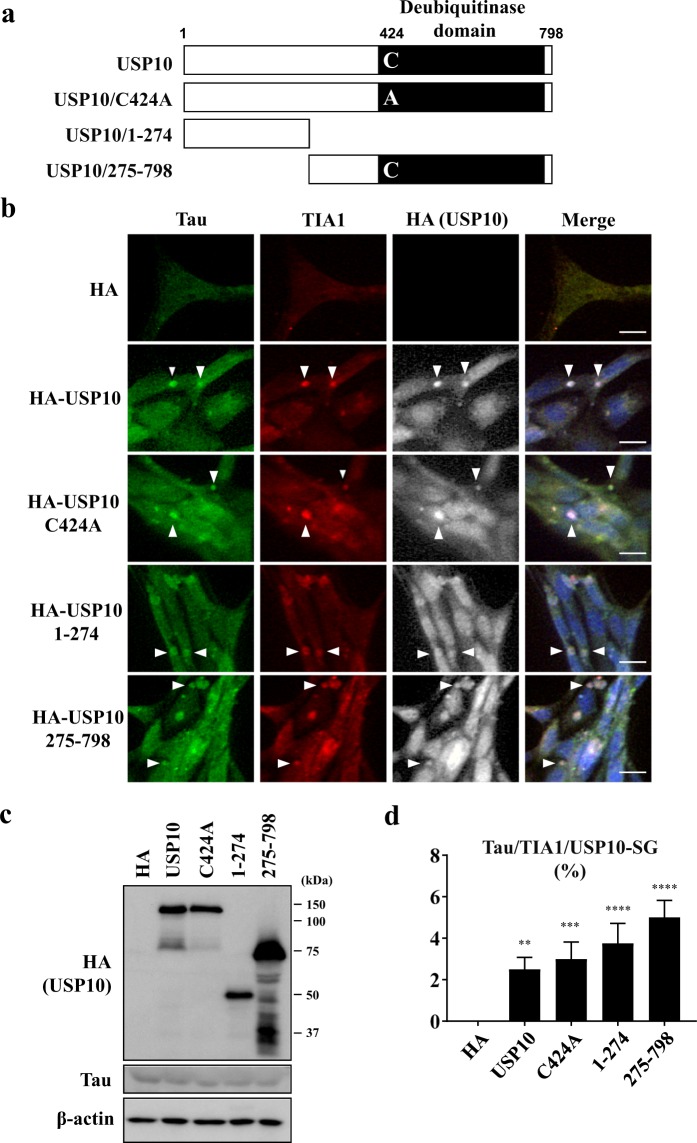
The deubiquitinase activity of USP10 is dispensable for Tau-SG formation. (**a**) A schematic illustration of USP10 and its mutants. (**b**–**d**) HT22 cells were transfected with a plasmid encoding HA-USP10 or its mutants (HA-USP10^C424A^, HA-USP10^1–274^, HA- USP10^275–798^) by lipofection using Lipofectamine 2000. At 48 h after transfection, SG formation was evaluated by anti-Tau (TAU-5), anti-TIA1 and anti-HA antibodies using a fluorescent microscope. Arrowheads indicate the colocalization of Tau, TIA1 and USP10. The population (%) of cells with Tau/TIA1/USP10-SGs is presented as the mean and SD from three experiments in (**d**). **P < 0.01; ***P < 0.001; ****P < 0.0001. Scale bar: 10 µm. (**c**) The expression of USP10 and its mutants, Tau and β-actin in these cells was measured by Western blotting using anti-HA, anti-Tau (TAU-5) and anti-β-actin antibodies, respectively.

To further examine the role of USP10 in Tau/TIA1/USP10-SG formation, HT22 cells were transfected with the plasmid encoding HA-tagged USP10 (HA-USP10) (Fig. 4). The overexpression of HA-USP10 without MG-132 treatment induced Tau/TIA1-SG formation, and these SGs were colocalized with HA-USP10. Through this experiment, we characterized USP10 mutants (Fig. 4a). USP10 has a deubiquitinase activity for several substrates, and USP10^C424A^ is the deubiquitination defective mutant^23,24^. USP10^C424A^ induced Tau-positive SGs with an activity equivalent to that of wild type USP10 (USP10-WT) (Fig. 4b-d), indicating that deubiquitinase activity of USP10 is dispensable for Tau/TIA1-SG formation. USP10^1–274^ and USP10^275–798^ were C- and N-terminal USP10 deletion mutants, respectively. Both USP10^1–274^ and USP10^275–798^ induced Tau-positive SGs at levels equivalent to that of USP10-WT (Fig. 4b–d). These results suggested that two regions of USP10 (USP10^1–274^ and USP10^275–798^) independently induce Tau/TIA1/USP10-SG formation.

**Figure 5:**
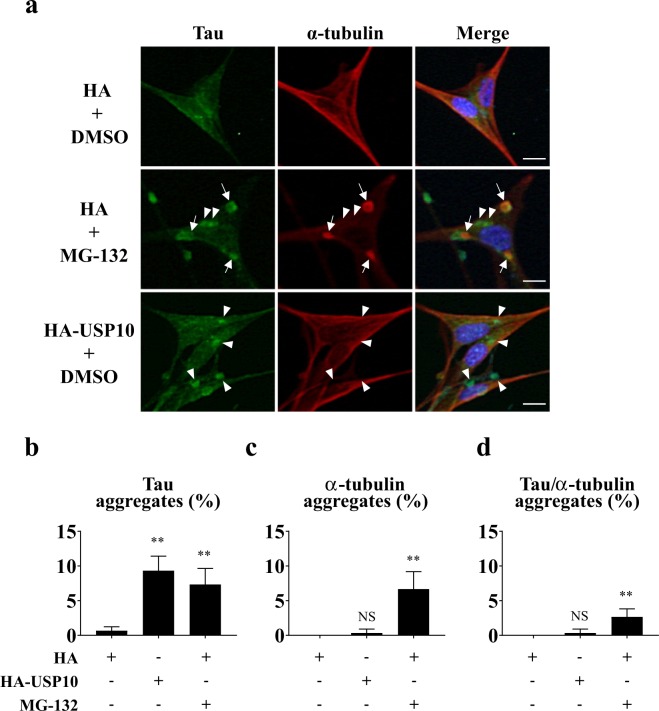
MG-132 treatment disrupts the microtubule structure in HT22 cells. (**a**–**d**) HT22 cells were transfected with the HA-USP10 plasmid or control plasmid (HA) by the lipofection method using Lipofectamine 2000. At 48 h after transfection, cells were treated with 5 µM MG-132 or DMSO for 4 h. The localization of Tau and α-tubulin was evaluated by anti-Tau (TAU-5) and anti-α-tubulin antibodies as well as Hoechst using a fluorescence microscope. Arrows and arrowheads indicate Tau/α-tubulin aggregates and Tau aggregates, respectively. Cells with either Tau aggregates, α-tubulin aggregates or Tau/α-tubulin aggregates were counted, and the respective population (%) is presented as the mean and SD from three independent experiments in (**b**–**d**). **P < 0.01. NS indicates that a result is not statistically significant. Scale bar: 10 µm.

Tau is a microtubule binding protein that stabilizes microtubules by binding to a heterodimer of α-tubulin and β-tubulin^31^. To gain information how Tau forms SGs in HT22 cells, we examined the localization of Tau and α-tubulin in HT22 cells. Before MG-132 treatment, α-tubulin showed fiber-like localization in HT22 cells (Fig. 5a), and the line profile analysis suggested that Tau are colocalized with these α-tubulin-positive fiber-like structure, especially near the cell membrane (Supplementary Fig. S4). However, these fiber-like localizations of α-tubulin were disrupted by MG-132 treatment, and α-tubulin-positive aggregates were induced (Fig. 5a). MG-132 treatment induced Tau-aggregates and some Tau-aggregates were partially colocalized with α-tubulin aggregates (Fig. 5b–d). These results suggested that the disruption of α-tubulin-positive microtubule structure by MG-132 plays a role in Tau-SGs formation. On the other hand, the overexpression of USP10 little reduced the fiber-like α-tubulin localization and little induced α-tubulin-aggregation (Fig. 5). These results suggested that the overexpression of USP10 induces Tau-SG formation in a mechanism with a distinction from that of MG-132.

Several SG-inducing agents, such as sodium arsenite treatment or the overexpression of G3BP1 induce the phosphorylation of eukaryotic initiation factor 2α (p-eIF2α), and the phosphorylation is associated with SG formation^32,33^. To evaluate the role of p-eIF2α in TIA1-induced SG formation, we measured the amount of p-eIF2α in HT22 cells transfected with either a G3BP1 or TIA1 plasmid. The overexpression of G3BP1 or TIA1 in HT22 cells little affected the amount of p-eIF2α (Supplementary Fig. S5). In addition, MG-132 treatment of HT22 cells only weakly increased the amount of p-eIF2α. These results suggested that p-eIF2α has little or no involvement in TIA1- or MG-132-induced SG formation in HT22 cells.

### Pathogenic TIA1 mutations augment Tau-positive SG formation

**Figure 6:**
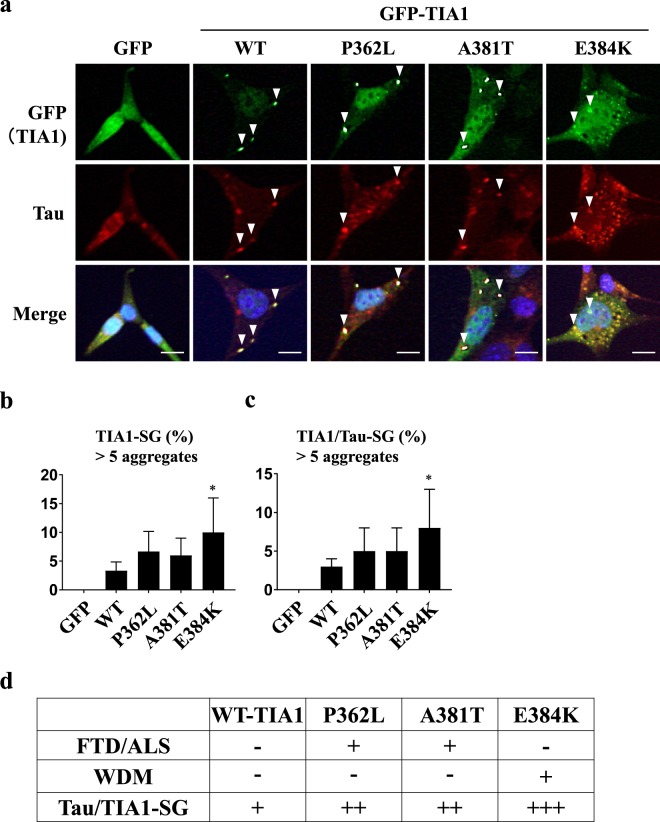
TIA1 pathogenic mutants augment Tau-SG formation. (**a**–**c**) HT22 cells were transfected with GFP-TIA1 or its mutant (GFP-TIA1/P362L, GFP-TIA1/A381T or GFP-TIA1/E384K). At 48 h after transfection, SG formation was evaluated by GFP fluorescence and anti-Tau (TAU-5) staining. Nuclei were stained with Hoechst. Arrowheads indicate the colocalization of GFP-TIA1 and Tau. The population (%) of cells with Tau/TIA1-SGs is presented as the mean and SD from three independent experiments in (**b**,**c**). *P < 0.05. Scale bar: 10 µm. (**d**) The SG-forming activities of the TIA1 mutants measured in (**a**–**c**) are summarized. The associations of TIA1 mutants with FTD/ALS and Welander Distal Myopathy (WDM) were reported in previous studies^34,35^. The association of WDM with TIA1/P362L or TIA1/A381T has not been reported^34^.

TIA1 mutations are associated with three diseases: frontotemporal dementia (FTD), amyotrophic lateral sclerosis (ALS) and Welander distal myopathy (WDM)^34–36^. FTD is a frequent cause of dementia characterized by the neuronal loss of the frontal and temporal lobes. ALS is a fatal neurodegenerative disease characterized by motor neuron loss. WDM is characterized by muscle atrophy of the distal upper extremities. Although these three diseases present with different clinical manifestations, a recent study suggested that FTD, ALS and WDM have related causative mechanism, such as perhaps TIA1 mutations^37–39^. Mackenzie *et al*. showed that TIA1 mutations derived from FTD/ALS or WDM patients induced augmented SG formation^35^. We examined whether or not disease-associated TIA1 mutations induce Tau-positive SGs. WDM-associated mutant TIA1/E384K induced significantly greater Tau/TIA1-SG formation than TIA1-WT (Fig. 6a–d). In addition, two FTD/ALS-associated TIA1 mutants (P362L, A381T) showed more marked Tau-positive SG-inducing activities than TIA1-WT, although the difference was statistically insignificant. These results suggested that Tau/TIA1-SGs play a role in the pathogenic activity in WDM, FTD and ALS.

### USP10 does not interact with Tau protein

**Figure 7:**
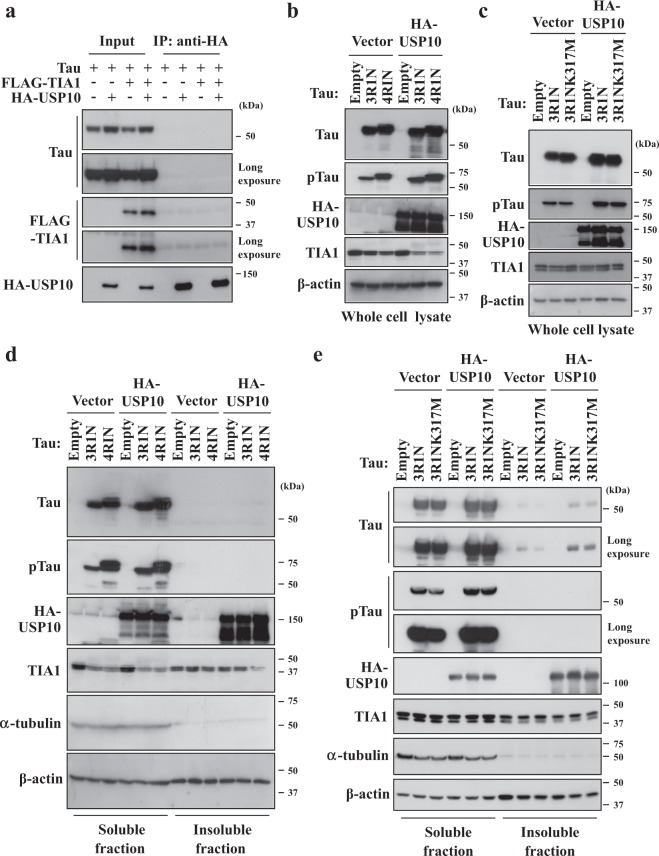
USP10 does not interact with Tau. (**a**) HT22 cells were transfected with Tau (Tau4R1N) plasmid along with HA-USP10 and/or FLAG-TIA1 plasmid using Lipofectamine 2000. Prepared cell lysates were immunoprecipitated with an anti-HA antibody, and the amounts of Tau, FLAG-TIA1 and HA-USP10 were analyzed by Western blotting using respective antibodies. (**b**–**e**) HeLa cells were transfected with HA-USP10 plasmid along either with Tau3R1N, Tau4R1N or Tau3R1N/K317M plasmid by lipofection using Lipofectamine 2000. At 48 h after transfection, whole cell lysates (**b**,**c**), NP-40-soluble fractions and NP-40-insoluble fractions (**d**,**e**) were prepared and characterized by Western blotting using anti-Tau (TAU-5), anti-phosphorylated Tau (pTau) (AT8), anti-HA, anti-TIA1, anti-α-tubulin and anti-β-actin antibodies.

A previous study showed that Tau interacts with TIA1 in HT22 cells. We therefore next examined whether USP10 interacts with Tau or TIA1 in HT22 cells. There are six tau isoforms in the human brain that are generated by alternative splicings: either zero, one or two amino-terminal inserts (0N, 1N, or 2N) and either three or four repeats of microtubule-binding domains (3R, 4R). A Tau plasmid (Tau4R1N) was transfected into HT22 cells along with HA-USP10 and/or a FLAG-tagged TIA1 plasmid (FLAG-TIA1) (Fig. 7a). An immunoprecipitation assay did not detect the interaction of USP10 with either Tau or TIA1. These results suggested that USP10 regulates Tau/TIA1/USP10-SG formation without binding strongly to Tau or TIA1 in HT22 cells.

### USP10 increases the amount of phosphorylated Tau protein

Mutations and hyperphosphorylation of Tau are associated with Tau aggregation in AD^3^. To gain further information on how USP10 promotes SG formation, we examined whether or not USP10 overexpression affects the amount and/or phosphorylation of Tau protein. The co-expression of USP10 either with Tau (Tau3R1N, Tau4R1N) in HeLa cells increased the amounts of Tau and phosphorylated Tau (pTau) detected with AT8 antibody (Fig. 7b). TauK317M is a pathogenic Tau mutant derived from frontotemporal dementia and parkinsonism linked to chromosome 17 (FTDP-17)^40^. USP10 also increased the amount of TauK317M protein to a level equivalent to that of Tau-WT (Fig. 7c). Cell fractionation with a detergent (NP-40) indicated that Tau (Tau4R1N, Tau3R1N, Tau3R1N/K317M) is expressed more in the soluble fraction than the insoluble fraction, and the amounts of both the soluble and insoluble Tau proteins were increased by USP10 co-expression (Fig. 7d,e). Given that hyperphosphorylation of Tau induces the separation from microtubules and promotes aggregation, these results suggested that USP10-induced Tau/TIA1/USP10-SG formation might be partly due to the increased amount of pTau protein. It is noteworthy that while a soluble Tau was predominantly detected in HeLa cells transfected with Tau plasmid (Fig. 7d,e), an insoluble Tau was predominantly detected in HT22 cells (Supplementary Fig. S3). Thus, distinct Tau localization in HeLa and HT22 cells might alter aspects of Tau activity, such as SG formation, in HeLa and HT22 cells, although the localization of an exogenous Tau protein in HT22 cells has not yet been chracterized. In addition, we detected (several times) the reduction of the TIA1 expression in HeLa cells transfected with the Tau plasmid (Fig. 7b,d). Thus, Tau might regulate the expression or degradation of TIA1 under certain conditions.

### USP10 is colocalized with pTau protein at Tau inclusions in AD brain

**Figure 8:**
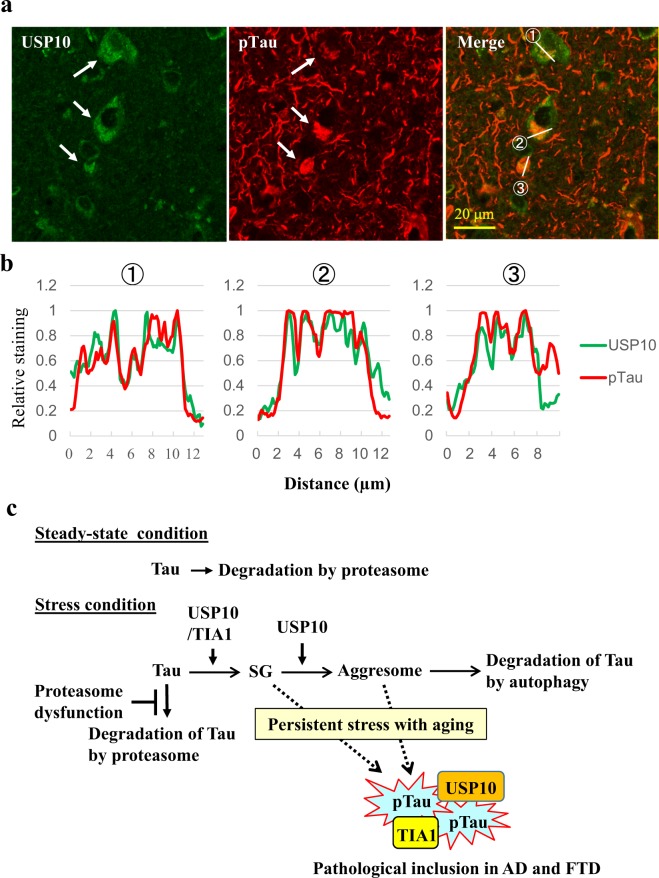
The colocalization of USP10 with pTau aggregates in AD brain regions. (**a**,**b**) Representative immunohistochemical findings of USP10 and pTau in temporal cortex of AD patients (n = 3) are shown. The colocalization of USP10 with pTau aggregates was analyzed by a line profile analysis. Arrows indicate the colocalization suggested by the assay. The relative staining levels of USP10 and pTau on the indicated line were presented (**b**). Lower magnification pictures were presented in Supplementary Fig. S6. (**c**) The current working model for pathogenic Tau aggregation through SGs and aggresomes.

pTau-positive inclusions in brain lesions are a hallmark pathology of AD. We examined the localization of USP10 and pTau (detected with AT8 antibody) in AD brain lesions (Fig. 8a,b, Supplementary Fig. S6). Immunohistochemical staining detected pTau-positive inclusions in cell bodies and neurites of the neurons of AD brains. USP10 was predominantly detected in cell bodies of most neurons and the line profile analysis suggested that some USP10 in cell bodies are colocalized with pTau inclusions (Fig. 8a,b). These results supported the notion that USP10 promotes pTau aggregation through SG formation in AD patients. Unlike pTau-inclusions in cell bodies, pTau-inclusions in neurites were not colocalized with USP10. These results are consistent with the finding that Tau/TIA1/USP10-SG is dominantly formed in cell bodies but not neurites (Figs 1–4).

## Discussion

Recent evidence has shown that TIA1-induced Tau-positive SG formation plays a critical role in Tau aggregation and Tau neurotoxicity in AD pathogenesis^14,15,35^. For instance, Vanderweyde *et al*. showed that TIA1 and Tau cooperatively induce Tau/TIA1-SG formation in cultured neuronal cells, and the formation was associated with Tau aggregation and neurotoxicity^14^. The reduction of TIA1 in AD model mice reduces pTau aggregation and Tau-induced neurotoxicity^15^. In the present study, we found that USP10 plays a key role in Tau/TIA1/USP10-SG formation in cultured neuronal cells (Figs 1–3). Furthermore, USP10 was colocalized with pTau-positive inclusions in brain lesion of AD (Fig. 8a,b). Therefore, the present study suggested that USP10 and TIA1 cooperatively promote pTau aggregation in AD through SG formation.

USP10 mutants revealed that the deubiquitinase activity of USP10 is dispensable for the SG-inducing activity and that both N- and C-terminal regions of USP10 (USP10^1–274^ and USP10^275–798^) have separate SG-inducing activities (Fig. 4b–d). We previously showed that both USP10^1–274^ and USP10^275–798^ mutants independently interact with the ubiquitin receptor p62^25^. Given that Tau is a ubiquitinated protein, these results suggest that p62 might play a role in the USP10-induced Tau-SG formation.

Tau is a microtubule binding protein^31^. The α-tubulin staining experiment suggested that MG-132 treatment induces SG formation partly through the disruption of microtubule structures (Fig. 5). On the other hand, while the overexpression of USP10 did not disrupt the microtubule structures, it increased the amount of pTau (Fig. 7b,c). The binding of Tau to microtubules is attenuated by the phosphorylation of Tau at multiple sites^3,4^. Thus, pTau is more easily released from microtubules than non-phosphorylated Tau and is efficiently recruited into SG. pTau increased by USP10 might therefore play a role in USP10-induced Tau-SG formation.

Our immunoprecipitation study failed to detect the interaction of USP10 with either TIA1 or Tau (Fig. 7a). However, Sowa *et al*. detected TIA1 as a binding protein of USP10^41^. Thus, a high-sensitivity binding experiment might detect the interaction of USP10 with either TIA1 or Tau in neuronal cells. Further analyses are needed regarding how USP10 induces TIA1/Tau-positive SG formation.

TIA1 mutations are associated with FTD, ALS and WDM^34,35^. We found that the WDM-associated TIA1 mutant E384K showed augmented Tau/TIA1-SG-inducing activity (Fig. 6a–c). Hackman *et al*. found that WDM patients with TIA1/E384K mutation had cytoplasmic TIA1-positive SG-like aggregates in atrophic muscle^34^. Thus, the present findings suggest that augmented Tau/TIA1-SG formation plays a role in WDM pathology. In addition, two FTD/ALS-associated TIA1 mutants (P362L, A381T) showed augmented SG-inducing activities relative to TIA1-WT, although the augmentation was statistically insignificant (Fig. 6b,c). Mackenzie *et al*. showed that the recombinant proteins of the same three TIA1 mutants characterized here induce greater protein aggregation (phase separation) activity than TIA1-WT protein. They also showed that these three TIA1 mutants showed more persistent SG formation after arsenite-treatment than TIA1-WT^35^. Given that Tau is one of the main causes of FTD^41^, these results suggest that augmented Tau/TIA1-SG formation induced by FTD/ALS-linked TIA1 mutations plays a role in the pathogenesis of FTD/ALS.

Aggresomes are stress-inducible protein aggregates containing many ubiquitinated neuropathogenic proteins, including Tau. MG-132 treatment induced the localization of Tau initially in SGs and then in aggresomes in HT22 cells (Fig. 1, Supplementary Fig. 2). Tau-positive aggresomes are colocalized with USP10 and TIA1 (Supplementary Fig. 2). In addition to SG formation, USP10 is a critical factor for aggresome formation. Taken together, these results suggested that USP10 and TIA1 cooperatively promote Tau-aggregation through the sequential localization of Tau in SG and aggresome (Fig. 8c). It should be noted that, under transient stress conditions, aggresome-localizing proteins are degraded by autophagy^5^ (Fig. 8c). However, under conditions inducing the persistent dysfunction of both proteasomes and autophagy, such as aging and/or chronic oxidative stress, SGs- or aggresome-localizing proteins might form pathogenic aggregates, such as Tau inclusions in AD.

USP10 promotes the pathogenic aggregation of α-synuclein, a causative factor of Parkinson’s disease (PD)^25^. PD is a neurodegenerative disease with impaired motor and cognitive functions characterized by α-synuclein aggregations known as Lewy bodies in affected brain lesions. While USP10 promotes α-synuclein aggregation and α-synuclein-positive aggresome formation in cultured cells, USP10 is localized in Lewy bodies with α-synuclein in PD brain samples. These results suggest that USP10, through SGs and/or aggresomes, promote the aggregation of multiple neuropathogenic proteins in several distinct neurodegenerative diseases.

## Methods

### Cells and culture

HT22 is a mouse hippocampal neuronal cell line^28^, and cells were cultured in Dulbecco’s modified Eagle medium (DMEM) supplemented with 15% heat-inactivated fetal bovine serum (FBS) and antibiotics at 37 °C under 5% CO_2_. HeLa cells were cultured in DMEM supplemented with 10% heat-inactivated FBS, 4 mM L-glutamine and antibiotics at 37 °C under 5% CO_2_.

### Preparation of primary rat neuron-enriched cells

Sprague-Dawley rats (Japan SLC, Inc., Shizuoka, Japan) were maintained in the animal care facility of the Niigata University Brain Research Institute. The experiment using rats was approved by the Animal Use and Care Committee of Niigata University and was carried out in accordance with the institutional guidelines and the guidelines of the National Institutes of Health Guide for the Care and Use of Laboratory Animals (NIH Publications No. 80–23).

Rat primary neuron-enriched cells were prepared as described previously^42^. Cortical tissues were dissected from rat embryos at embryonic days 16–19 and digested with papain (Worthington Biochemical Corporation, Lakewood, NJ). Cells were plated on glass coverslips coated with poly-D-lysine (Sigma-Aldrich, St. Louis, MO) in a 6-well plate (Corning, NY, USA) and cultured in DMEM supplemented with 10% FBS. After overnight culturing, the medium was replaced with serum-free DMEM supplemented with N-2 Supplement (Gibco, MA, USA) and L-glutamine, and then cells were further cultured for 3 d. Cells were then treated with 2 µM MG-132 or DMSO for 8 h and characterized for SG formation by immunostaining.

### Establishment of USP10-knockdown cells

Knockdown of endogenous USP10 in HT22 cells was performed using the lentivirus vector pLKO.1-puro encoding USP10 shRNA. The three plasmids (pLKO.1-puro, pCMV-VSV-G-RSV-Rev and pCAG-HIVgp) were introduced into 293 T cells using FuGENE HD (0000266475; Roche, Switzerland) according to the manufacturer’s instructions. At 72 h after the transfection, the supernatant was collected and infected to HT22 cells in the presence of 8 μg/μl polybrene. At 48 h after the infection, cells were cultured in the medium containing 4 μg/ml puromycin. Knockdown efficiency was evaluated by Western blotting.

### Reagents and antibodies

MG-132 (474790; Calbiochem, Cambridge, MA), puromycin (P8833; Sigma-Aldrich) and Hoechst 33258 (H-3569; Molecular Probes, Eugene, OR) were purchased from the indicated companies. The following antibodies were used in this study: rabbit anti-Tau (sc-5587; Santa Cruz Biotechnology, Santa Cruz, CA), mouse anti-Tau (TAU-5) (AHB 0042; Invitrogen, Carlsbad, CA), mouse anti-phosphorylated Tau (AT8) that recognizes phosphorylated Ser202/Thr205 of human Tau (MN1020; Invitrogen), goat anti-TIA1 (sc-1751; Santa Cruz Biotechnology), rabbit anti-TIA1 (ab40693; Abcam), rabbit anti-PABP (GR2766925-1, Abcam), mouse anti-eIF4E (610269; BD Transduction Laboratories, San Jose, CA), mouse anti-ataxin-2 (611378; BD Transduction Laboratories), rabbit anti-USP10 (A300-901A; Bethyl Laboratories, Montgomery, TX; HPA006731; Sigma-Aldrich), mouse anti-G3BP (611127; BD Transduction Laboratories), mouse anti-α-tubulin (D00028259; Calbiochem), rabbit anti-α-tubulin (GR3190631-1, Abcam), rabbit anti-eIF2α (9722; Cell Signaling, Danvers, MA), rabbit anti-phosphorylated eIF2α (9721; Cell Signaling), mouse anti-HA (3724; Cell Signaling), mouse anti-FLAG (cloneM2, 087K60021; Sigma-Aldrich) and mouse anti-β-actin (sc-47778; Santa Cruz Biotechnology).

### Plasmids

The USP10 plasmids used were described previously^12^. Plasmids encoding USP10-WT or its mutants (USP10^C424A^, USP10^1–274^, USP10^275–798^) with an N-terminal hemagglutinin (HA) epitope tag were generated by subcloning the respective cDNAs into an expression plasmid pCMV-HA (Clontech)^12^. Tau-WT plasmids (Tau3R1N, Tau4R1N) and its mutant (Tau3R1N/K317M) were described previously^40^. Wild type GFP-TIA1 plasmid and its mutants (GFP-TIA1/P362L, GFP-TIA1/A381T and GFP-TIA1/E384K) were kindly provided by Dr. J. Paul Taylor (St. Jude Children’s Research Hospital, Memphis, TN)^35^. FLAG-tagged TIA1 plasmid (p3xFLAG-TIA1) was kindly provided by Dr. Ravindra Singh (Iowa State University, Ames, IA, USA)^43^.

### Plasmid transfection

HT22 (1.5 × 10^5^) or HeLa cells (1.5 × 10^5^) were seeded on a 6-well plate (Corning) 1 day before transfection. Cells were transfected with the plasmids with Lipofectamine 2000 (1910607; Invitrogen) in Opti-MEM (1869048; Life Technologies) according to the manufacturer’s instructions. Cells were used for the analysis at 48 h after transfection.

### Immunofluorescence analyses

The cells were plated on glass coverslips in 6-well plates, and then fixed with 3.7% formaldehyde in phosphate-buffered saline (PBS) for 15 min at room temperature and permeabilized by 0.1% Triton X-100 in PBS for 5 min at room temperature. The fixed cells were incubated with the primary antibody for 60 min at room temperature, washed with PBS, and then incubated with the secondary antibody and Hoechst 33258 for nuclear staining for 60 min at room temperature. The cell image was obtained using a fluorescence microscope (BZ-8000 or BZ-X800; KEYENCE, Osaka, Japan). The secondary antibodies used in this study were Alexa488-conjugated anti-rabbit or anti-mouse immunoglobulin, Alexa594-conjugated anti-rabbit, anti-mouse or anti-goat immunoglobulin and Alexa405-conjugated anti-rabbit immunoglobulin (Molecular Probes).

### SG formation

To quantify SG formation (Tau/TIA1-SG, Tau/USP10-SG, Tau/G3BP-SG, Tau/TIA1/USP10-SG, Tau/GFP-SG), cells were analysed by immunostaining with anti-Tau, anti-TIA1, anti-G3BP, anti-USP10 or anti-HA (HA-USP10). TIA1/Tau-positive-aggregates (more than 1 μm^2^ in size) were evaluated as TIA1/Tau-SGs. The same definition was used for Tau/TIA1-SGs, Tau/TIA1/USP10-SGs and G3BP-SGs. Cells with more than three SGs were counted as SG-positive cells. More than 100 cells per field from 4 random fields were used to calculate the average and standard deviation (SD), and the ratio of SG-positive cells to the total cells was presented.

### Western blotting

Cells were washed with PBS and lysed in sodium dodecylsulfate (SDS) lysis buffer (2% SDS, 62.5 mM Tris-HCI [pH 6.8], 10% glycerol and 50 mM dithiothreitol), and then sonicated and heated at 95 °C for 5 min and centrifuged for 10 min. After centrifugation, the protein amount in the supernatant was measured using a Bio-Rad DC^TM^ Protein assay Kit. About 100 µg of cell lysate was loaded onto an 8% polyacrylamide gel containing SDS, and electrophoresis was performed at 100 V for 2 h (PAGE). Gels were then transferred to a PVDF membrane. Membranes were blocked with 5% skim milk in TBST buffer (Tris-buffered saline, 0.2% Tween-20) and incubated with the primary antibody for 1 h at room temperature. Membranes were washed in TBST buffer and incubated with the secondary antibody conjugated to horseradish peroxidase for 1 h at room temperature, and antibody binding was visualized using an ECL Western blotting detection system (GE Healthcare, Chicago, IL).

### Immunoprecipitation assays

Plasmids were transfected into HT22 cells using Lipofectamine 2000. At 48 h after transfection, cells were lysed in cold NP-40 lysis buffer (1% Nonidet P-40, 125 mM Tris-HCl, pH 7.2, 150 mM NaCl, 1 mM EDTA, 1 mM phenylmethylsulfonyl fluoride, 0.2 µg/ml aprotinin), and cell lysates were immunoprecipitated with anti-HA antibody. The immunoprecipitated proteins were separated by PAGE, transferred to PVDF membranes, and characterized by Western blotting using anti-HA, anti-FLAG and anti-Tau antibody (TAU-5).

### Fractionation assay

Cells were lysed with cold NP-40 lysis buffer for 10 min. After centrifugation, the supernatant was collected as the NP-40-soluble fraction. The pellet was treated with SDS lysis buffer, and the supernatant was used as the NP-40-insoluble fraction. To prepare whole-cell lysates, cells were directly treated with the SDS lysis buffer, and the supernatants were used as whole-cell lysates. Soluble, insoluble and whole-cell lysates were analysed by Western blotting.

### Pathological analyses

The study using human samples was performed with the approval of the ethics committees of Niigata University (approval number: G2015-0686). Written informed consent for autopsy, collection of samples and subsequent use for research purposes was obtained from the next of kin of the deceased involved in this study. The autopsied brain tissues (temporal cortex) of AD patients (n = 3) and controls (n = 3) were used. Formalin-fixed, paraffin-embedded sections 4-μm-thick were immunostained using a rabbit polyclonal antibody against USP10 (HPA006731; Sigma-Aldrich; diluted 1:2,000, pretreated by heating) and a mouse monoclonal antibody against phosphorylated Tau (AT8; 90206; IGT; diluted 1:200). The primary antibody binding was detected by anti-immunoglobulin secondary antibodies conjugated with either Alexa488 or Alexa568 (respectively anti-rabbit or anti-mouse; Molecular Probes). Immunofluorescent imaging was observed by a confocal microscope (FV3000RS; Olympus, Tokyo, Japan).

### Colocalization assay

Colocalization of USP10 and pTau was examined by a line profile analysis. The staining levels of pTau and USP10 on the indicated line in immunohistochemical staining of AD samples were measured by the ImageJ software program. Relative staining of pTau and USP10 was calculated as a ratio of the respective staining level relative to the maximum level. Relative staining of pTau and USP10 is shown on the indicated lines from left to right. Colocalization of Tau and α-tubulin in HT22 cells was examined by the same method using a fluorescence microscope (BZ-X800).

### Statistical analyses

Statistical analyses were performed using the Student’s *t*-test or one-way analysis of variance followed by Tukey’s Multiple Comparisons Test using the Prism7 software program (GraphPad, San Diego, CA) and were presented as the mean and standard deviation (SD).

## Supplementary information

Supplementary Information

## Data Availability

The data that support the findings of this study are available from the corresponding author upon reasonable request.
