# Supplementary material for: USP10 is a critical factor for Tau-positive stress granule formation in neuronal cells
Source: Sci Rep. 2019 Jul 22;9:10591. doi: 10.1038/s41598-019-47033-7 (PMC6646309; doi:10.1038/s41598-019-47033-7)
Supplement: Supplementary file 1 — Supplementary Information [file 41598_2019_47033_MOESM1_ESM.pdf]

Supplementary Information

USP10 is a critical factor for Tau-positive stress granule formation in neuronal cells

Svetlana Piatnitskaia,[1,*] Masahiko Takahashi,[1,*] Hiroki Kitaura,[2] Yoshinori Katsuragi,[1] Taichi Kakihana,[1] Lu Zhang,[2] Akiyoshi Kakita,[2] Yuriko Iwakura,[3] Hiroyuki Nawa,[3] Takeshi Miura,[4] Takeshi Ikeuchi,[4] Toshifumi Hara,[5] Masahiro Fujii[1]

[1]Division of Virology, Niigata University Graduate School of Medical and Dental Sciences, Niigata, 951-8510, Japan
[2]Department of Pathology, Brain Research Institute, Niigata University, Niigata, 951-8585, Japan
[3]Department of Molecular Neurobiology, Brain Research Institute, Niigata University, Niigata, 951-8585, Japan
[4]Department of Molecular Genetics, Brain Research Institute, Niigata University, Niigata, 951-8585, Japan.
[5]Department of Medicinal Biochemistry, School of Pharmacy, Aichi Gakuin University, Nagoya, Japan.
[*]These two authors contributed equally to this study.
Correspondence and requests for materials should be addressed to M.F. (e-mail: fujiimas@med.niigata-u.ac.jp)

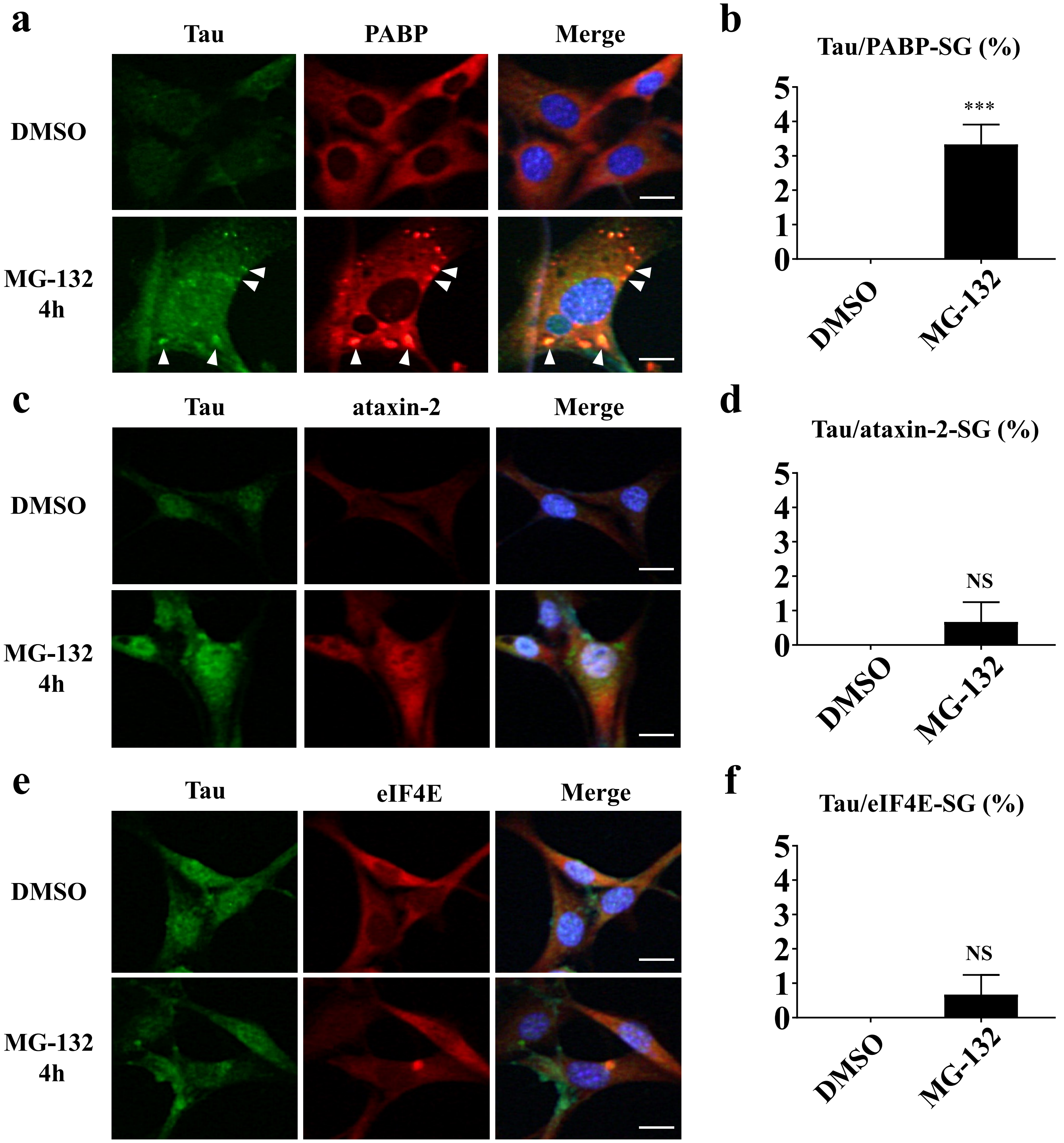

Supplementary Figure S1. PABP is colocalized with Tau-SGs in HT22 cells. (**a-f**) HT22 cells were treated with 5 μM MG-132 for 4 h and stained with anti-Tau (TAU-5) plus anti-PABP (**a, b**), anti-ataxin-2 (**c, d**) or anti-eIF4E (**e, f**). Nuclei were stained with Hoechst. Staining was visualized using a fluorescence microscope. Arrowheads indicate the colocalization of Tau with PABP (**a**). The populations (%) of cells with Tau/PABP-SGs, with Tau/ataxin-2-SGs, Tau/eIF4E-SGs are presented as the mean and SD from three independent experiments in (**b, d, f**). ***P <0.001. NS indicates that a result is not statistically significant. Scale bars: 10 μm.

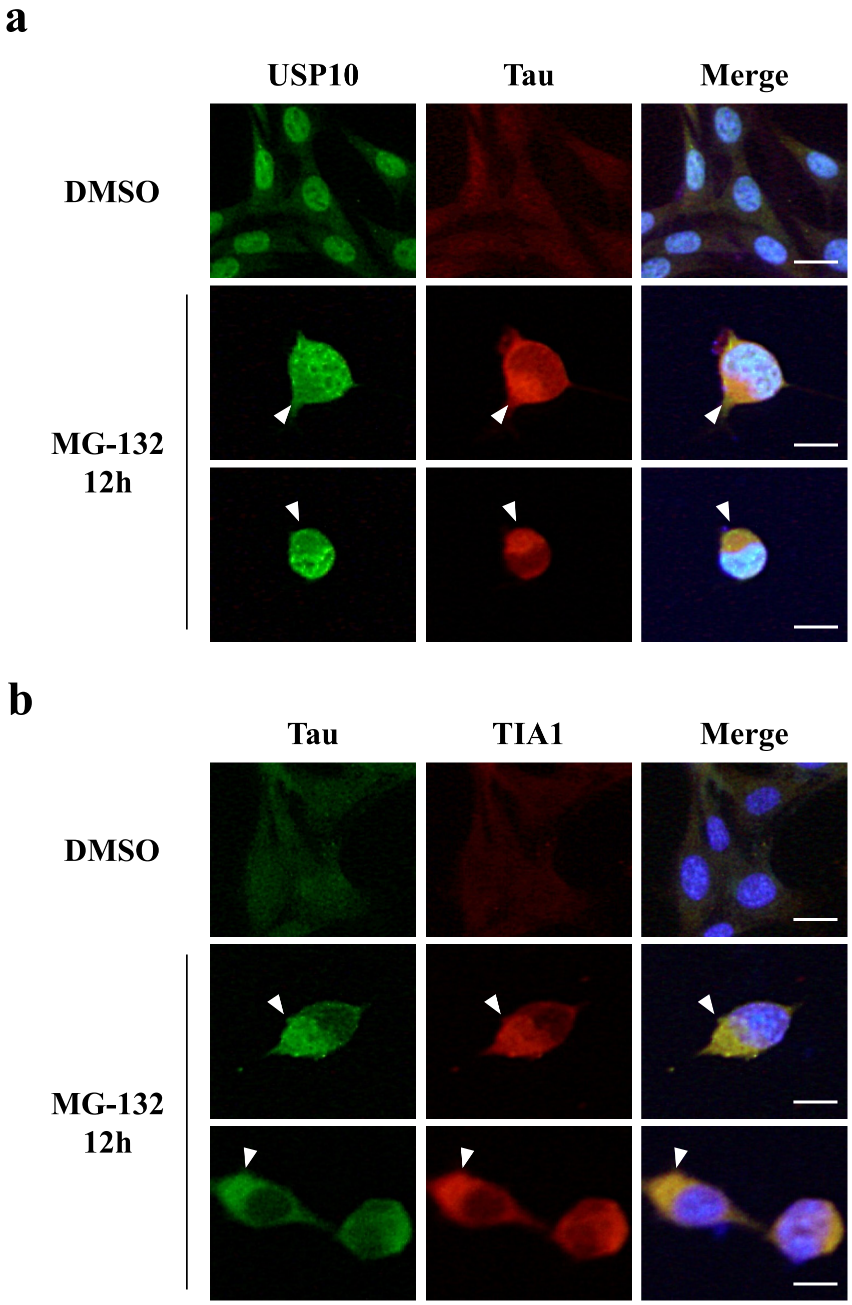

Supplementary Figure S2. Tau is localized in aggresomes in HT22 neuronal cells. (**a, b**) HT22 cells were treated with 5 μM MG-132 for 12 h and stained with anti-Tau (TAU-5) plus anti-USP10 or anti-Tau (TAU-5) plus anti-TIA1 antibodies. Nuclei were stained with Hoechst. SGs were visualized using a fluorescent microscope. Arrows indicate the colocalization of Tau with TIA1 or USP10. Scale bars: 10 μm.

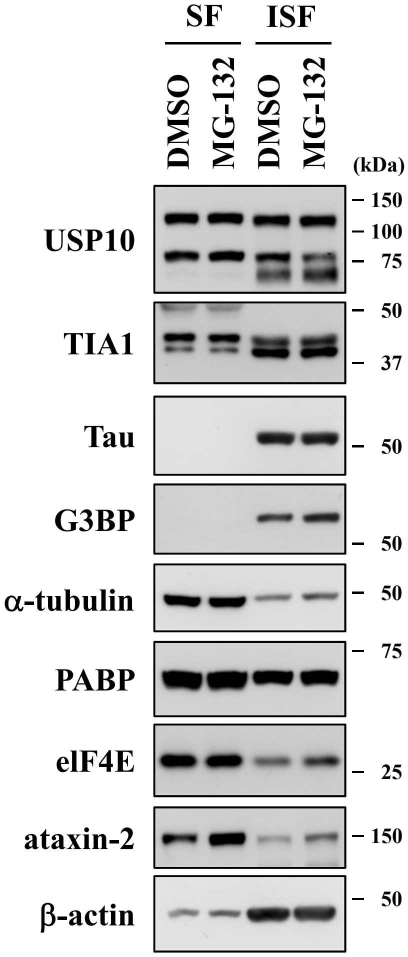

Supplementary Figure S3. The expression of SG-associated proteins in HT22 cells treated with MG-132. HT22 cells were treated with 5 μM MG-132 for 4 h. NP-40-soluble fractions (SF) and NP-40-insoluble fractions (ISF) were prepared from these cells and characterized by Western blotting using the indicated antibodies.

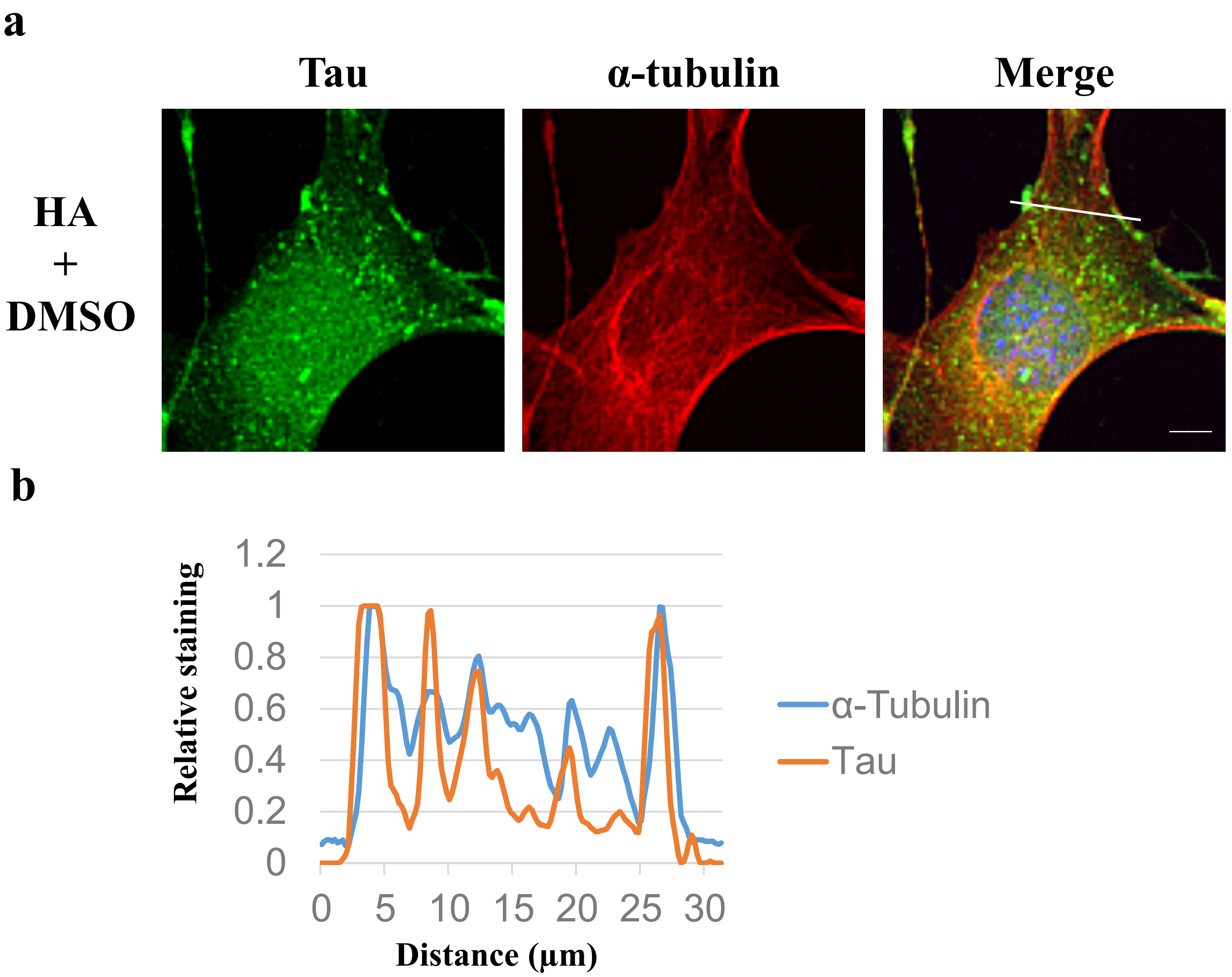

Supplementary Figure S4. The colocalization of Tau with α–tubulin-positive fiber-like structure in HT22 cells. (**a, b**) Representative immunostaining pictures of Tau and α-tubulin in HT22 cells. The colocalization of Tau with α-tubulin was analyzed by a line profile analysis. The relative staining levels of Tau and α-tubulin on the indicated line were presented in (**b**). Scale bars: 10 μm.

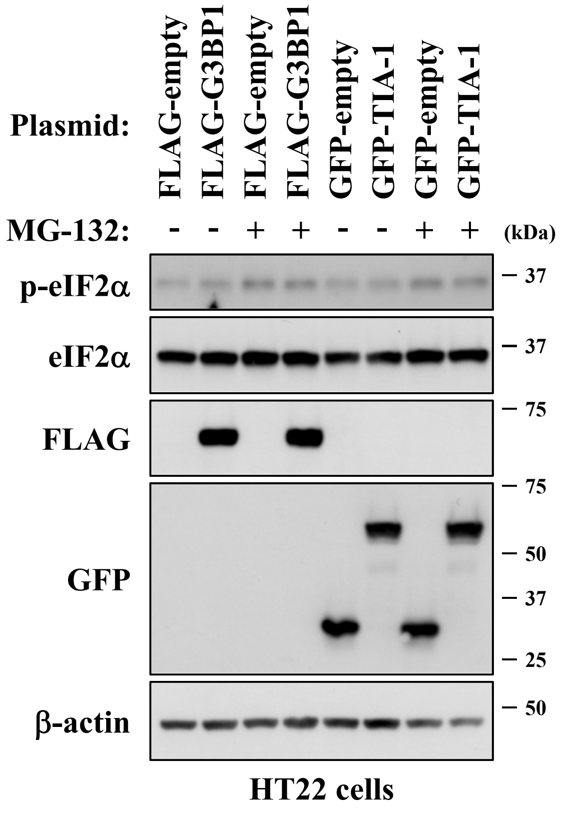

Supplementary Figure S5. The levels of eIF2 phosphorylation (p-eIF2α) in SG-positive HT22 cells. HT22 cells were transfected with FLAG-G3BP1, GFP-TIA1 or their control plasmids by lipofection using Lipofectamine 2000. At 48 h after transfection, cells were treated with MG-132 for 4 h. Whole cell lysates were prepared and characterized by Western blotting using indicated antibodies.

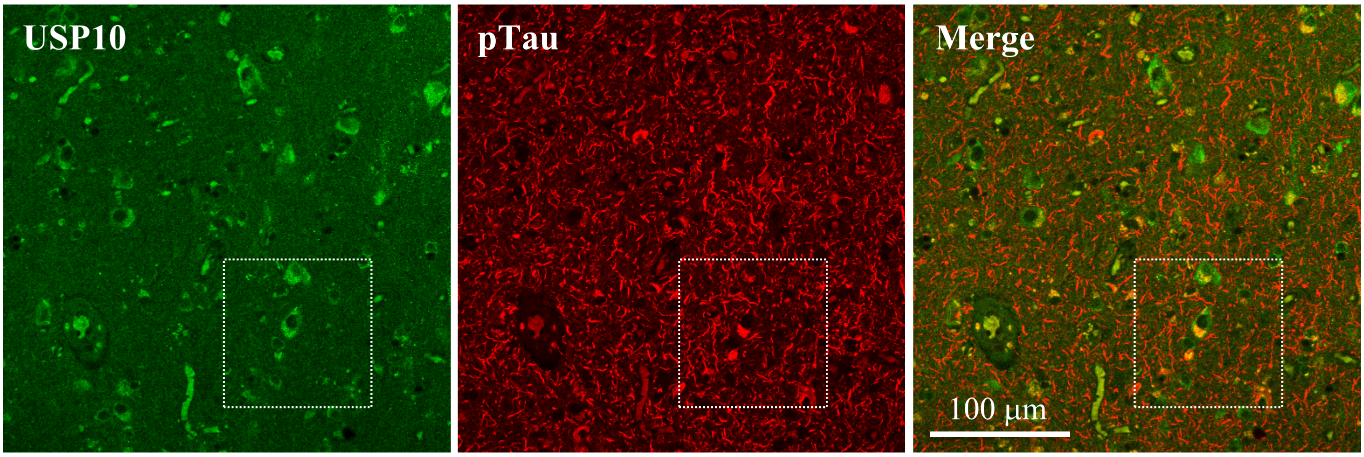

Supplementary Figure S6. The colocalization of USP10 with pTau aggregates in AD brain regions. Representative immunohistochemical findings of USP10 and pTau in temporal cortex of AD patients (n=3) are shown. Higher magnification pictures of the areas located in the squares are presented in Fig. 8a.

Figure 2a

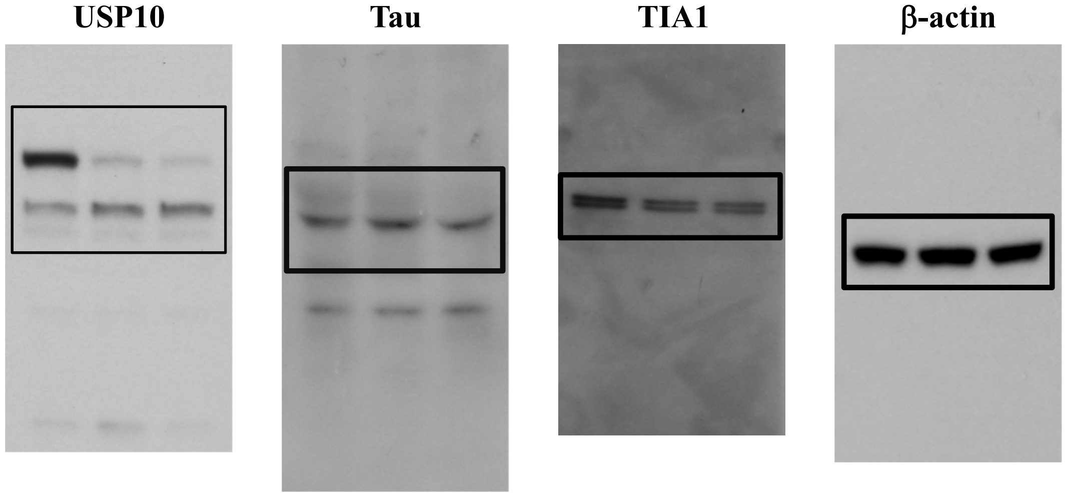

Supplementary Figure S7. Full length blots shown in Figure 2a.

Figure 4c

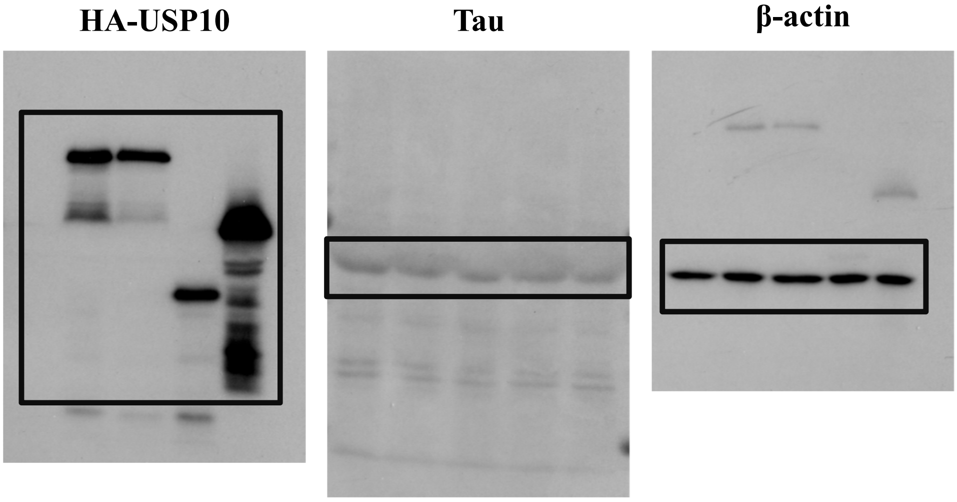

Supplementary Figure S8. Full length blots shown in Figure 4c.

Figure 7a

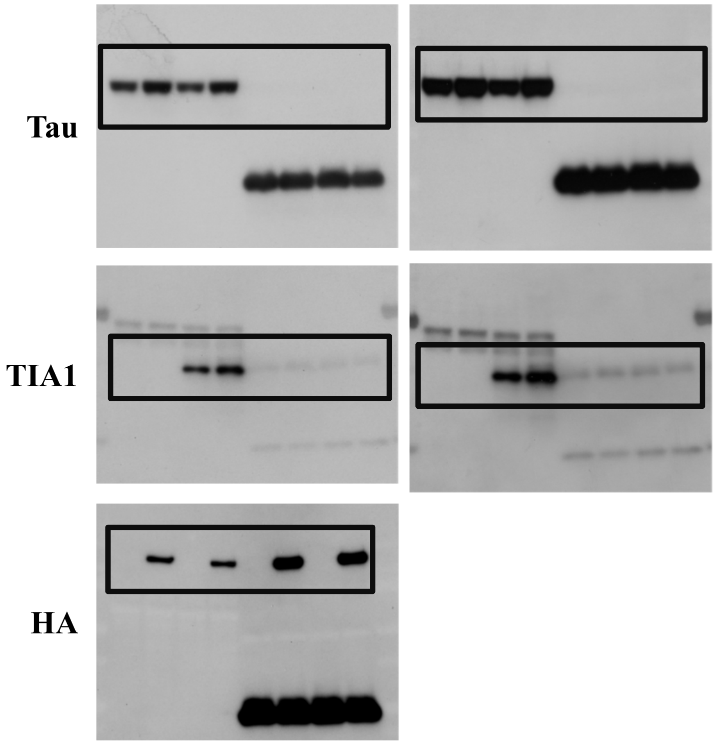

Figure 7b

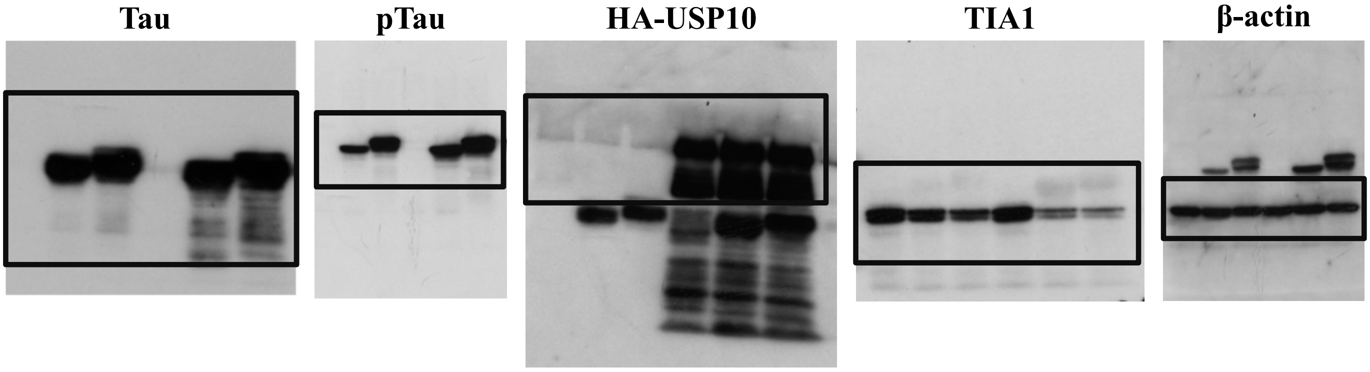

Figure 7c

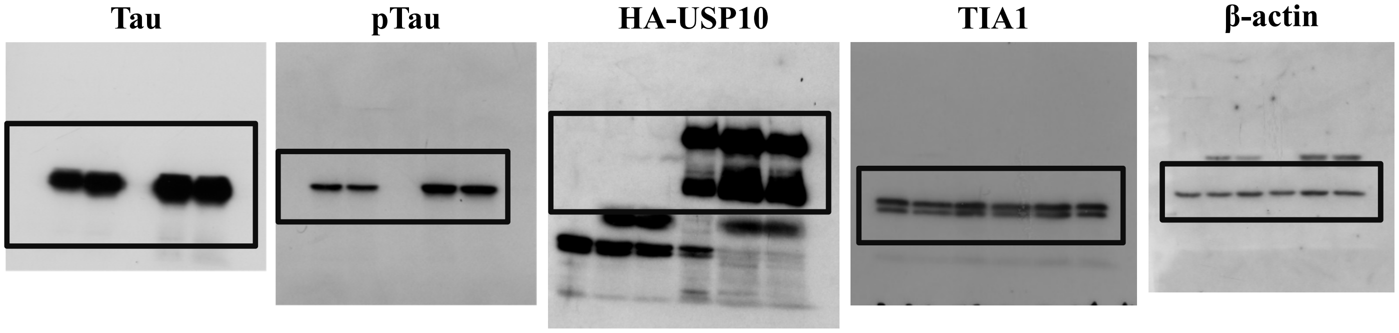

Supplementary Figure S9. Full length blots shown in Figure 7a-c.

Figure 7d

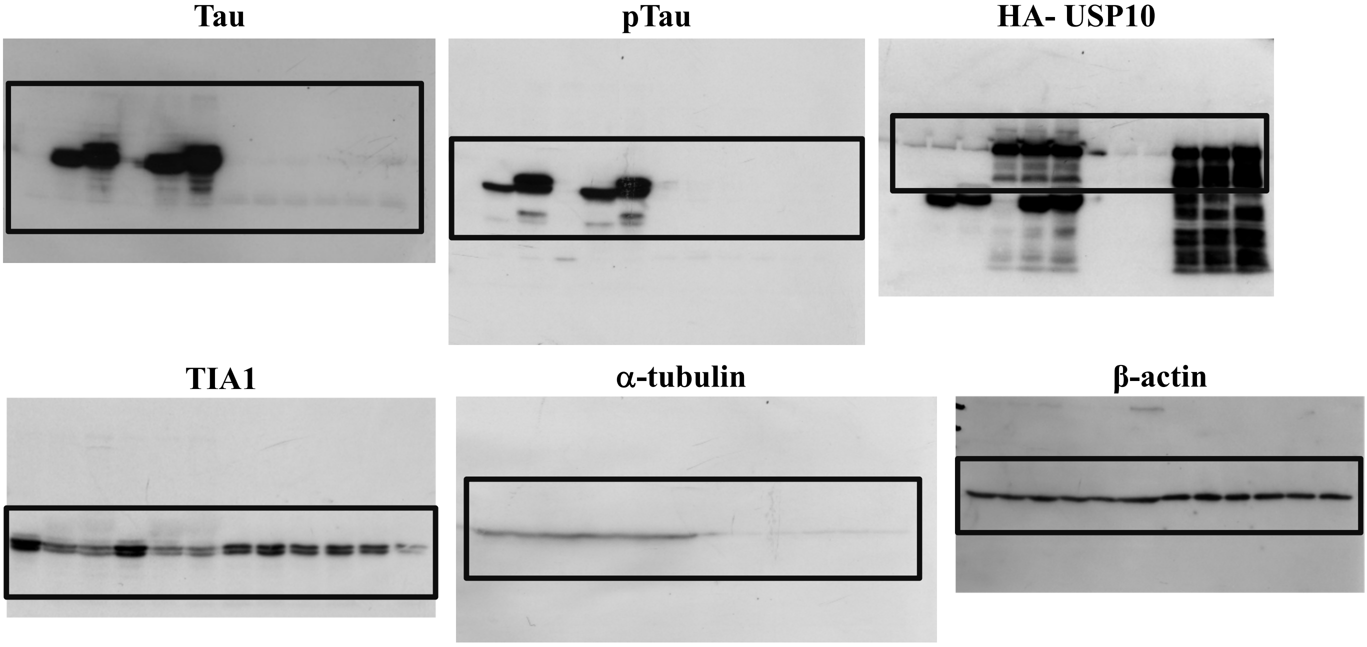

Figure 7e

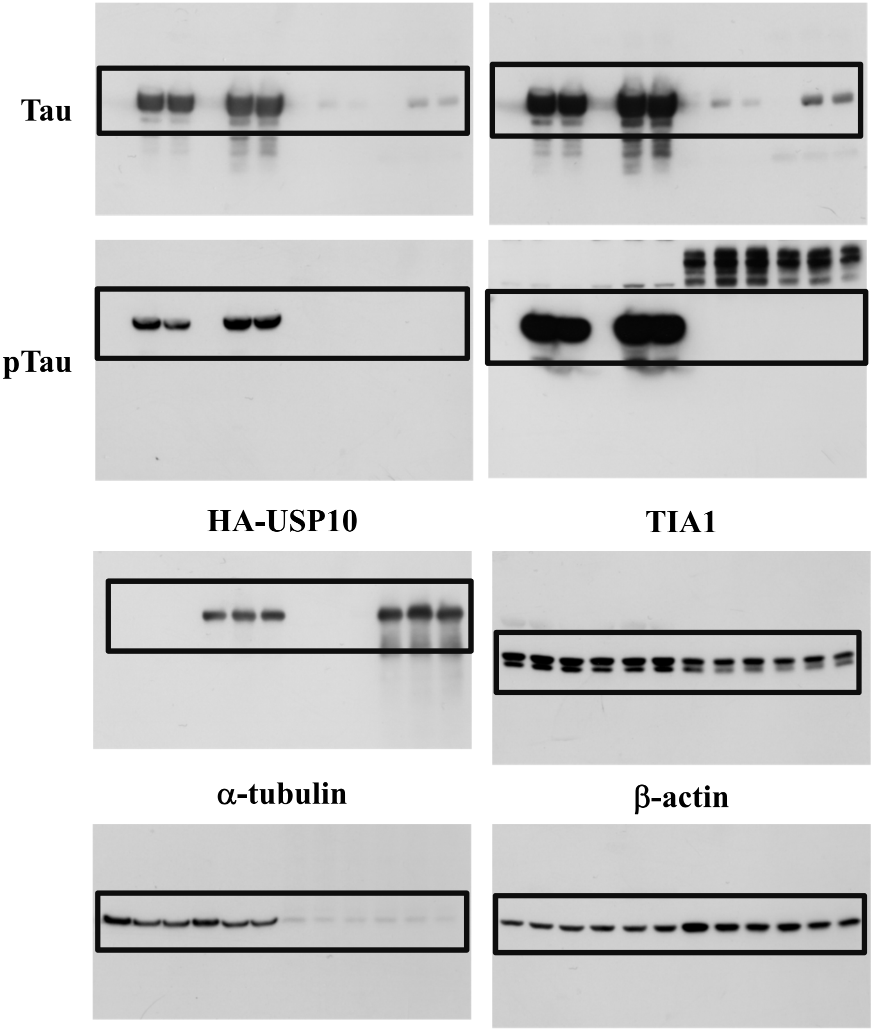

Supplementary Figure S10. Full length blots shown in Figure 7d, e.

Supplementary Figure S3

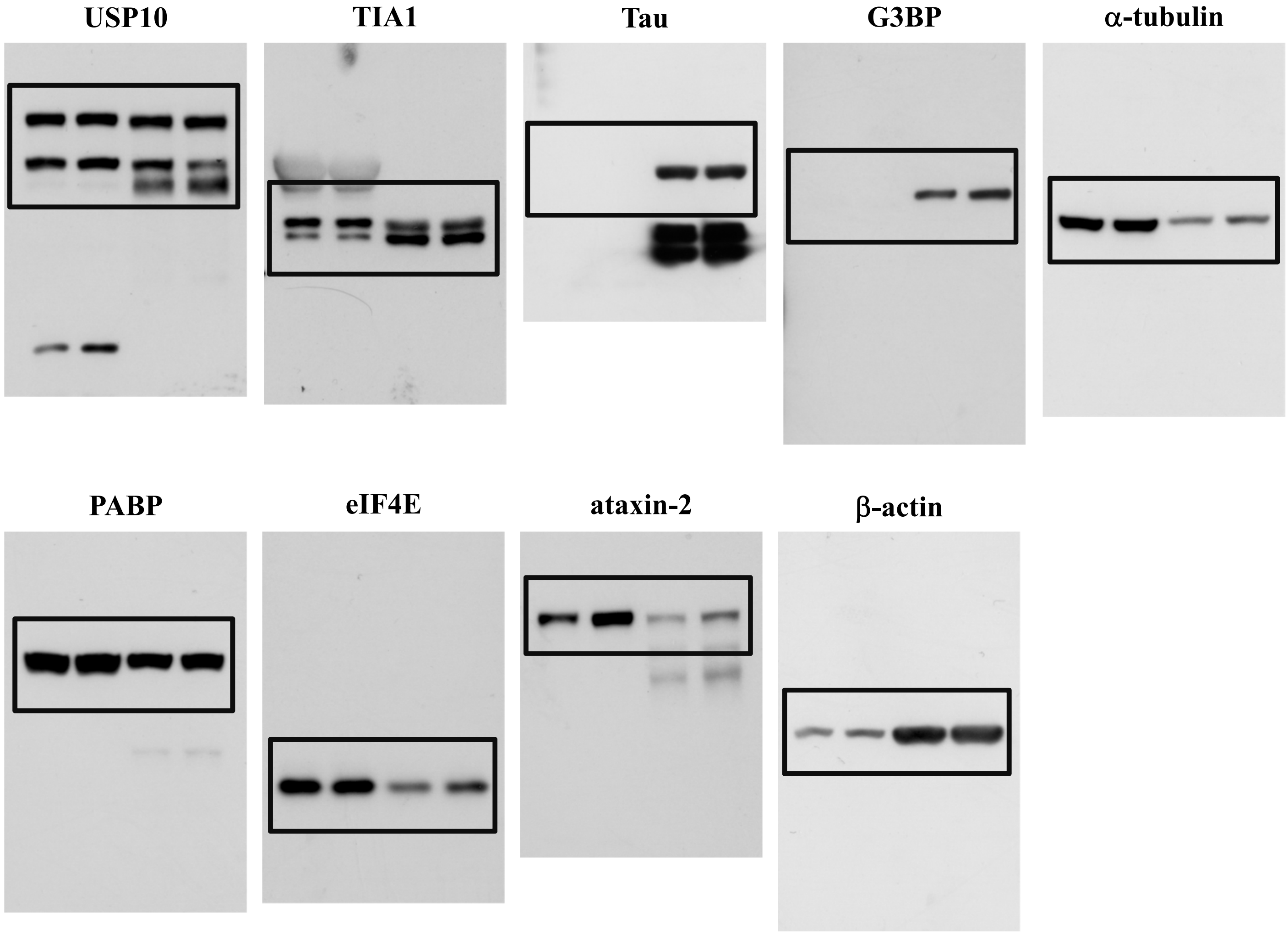

Supplementary Figure S11. Full length blots shown in Supplementary Figure S3.

Supplementary Figure S5

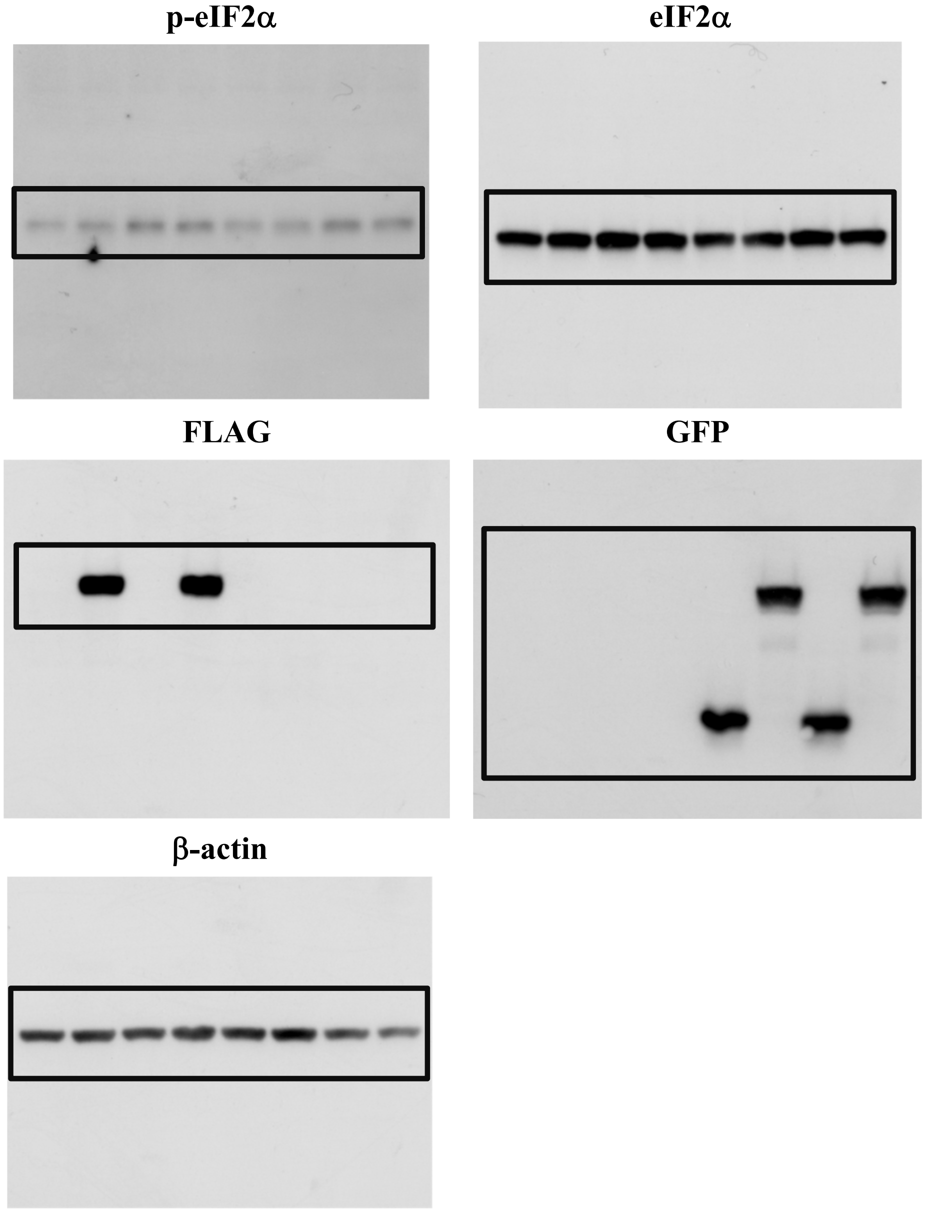

Supplementary Figure S12. Full length blots shown in Supplementary Figure S5.
